# The Effect of Thickness on the Sealing Ability of CEM Cement as a Root-end Filling Material

**DOI:** 10.15171/joddd.2015.002

**Published:** 2015-03-04

**Authors:** Saeed Rahimi, Saeed Asgary, Mohammad Samiei, Mahmoud Bahari, Seyyed Mahdi Vahid Pakdel, Rasoul Mahmoudi

**Affiliations:** ^1^Dental and Periodontal Research Center, Tabriz University of Medical Sciences, Tabriz, Iran; ^2^Professor, Department of Endodontics, School of Dentistry, Tabriz University of Medical Sciences, Tabriz, Iran; ^3^Iranian Center for Endodontic Research, Dental Research Center, Shahid Beheshti University of Medical Sciences, Tehran, Iran; ^4^Assistant professor, Department of Endodontics, School of Dentistry, Tabriz University of Medical Sciences, Tabriz, Iran; ^5^Assistant professor, Department of Operative Dentistry, School of Dentistry, Tabriz University of Medical Sciences, Tabriz, Iran; ^6^Post-graduate student, Department of Prosthodontics, School of Dentistry, Tabriz University of Medical Sciences, Tabriz, Iran; ^7^ Private Practice, Tabriz, Iran

**Keywords:** Calcium enriched mixture, CEM cement, Leakage, root-end filling materials, sealing efficacy

## Abstract

***Background and aims.*** Different materials have been used for root-end filling during surgical endodontic treatment. The aim of this *in vitro* study was to evaluate the dye penetration in different thicknesses of calcium enriched mixture (CEM) cement as root-end filling material.

*** Materials and methods.*** Following root canal filling in 70 extracted human single-rooted premolar teeth, the apical 3 mm of their root-ends was resected; the root-end cavities with depths of 1, 2 and 3 mm were prepared by ultrasonic retrotips and filled with CEM cement. After setting of cement, the roots were immersed in 2% Rhodamine B and the dye leakage was measured under stereomicroscope (×16) using Image J software. The data were analyzed by one-way ANOVA and Bonferroni *post hoc* tests at 5% significance level.

*** Results. ***The means and standard deviations of dye penetration in the 1, 2, and 3 mm groups were 3395.5±1893.4, 3410.4±1440.5, and 2581.6±1852.9 μm, respectively. The one-way ANOVA analysis indicated significant differences (P < 0.001); however, the Bonferroni *post hoc* test revealed that only the positive control group differed significantly from the experimental groups (P < 0.001).

*** Conclusion.*** The findings demonstrated CEM cement to have an adequate root-end sealing ability in 3-mm thickness.

## Introduction

Surgical endodontic retreatment is essential in cases of failed endodontic treatment or when conventional endodontic treatment cannot be undertaken. Endodontic surgery entails raising a mucoperiosteal flap and performing osteotomy, followed by root-end resection, root-end preparation, and root-end filling. A root-end filling material is considered effective when it provides a complete apical seal, preventing the passage of microorganisms into the root canal system and leading to the throwing out of microorganisms and their by-products from the root canal system.^1^

Various materials have been suggested and used for root end filling. They include zinc oxide eugenol cements, glass ionomer cement, super EBA, polyvinyl resins, composite resins, resin-glass ionomer hybrids, and mineral trioxide aggregate (MTA).^2-6^

Microleakage studies have confirmed MTA has the best apical sealing ability. However, despite its excellent sealing ability and biocompatibility when compared with other root-end filling materials, MTA is expensive, has delayed setting time, and poor handling properties.^7,8^ Asgary et al^9^ have introduced a novel endodontic cement with sealing ability comparable to MTA. This biomaterial was formulated using different calcium compounds such as calcium hydroxide, calcium oxide, calcium phosphate, calcium sulfate, calcium silicate, and calcium carbonate. Studies have demonstrated that the calcium-enriched mixture (CEM) cement comprises water-soluble calcium and phosphate which immediately forms hydroxyapatite during and after setting.^9^

The composition and surface characteristics as well as the physical and chemical properties of CEM cement have been examined. CEM cement has been shown to have a sealing ability comparable to MTA and superior to IRM.,^[Bibr R06],10^ The cement exhibits several advantages including high tissue biocompatibility, hard tissue induction, effective sealing ability against the entry of microorganisms, ability to set in an aqueous environment, antibacterial effects, and resistance to washout.^10-11,13^ *In-vitro* studies have documented the equivalence of CEM cement and MTA properties.^10,14,15^ *In-vivo* studies have also produced successful results with CEM cement.^11,12,16-20^

In an *in-vitro* study, the influence of the thickness of mineral trioxide aggregate on the sealing ability of root-end fillings was assessed by Valois et al^21^ The results revealed a thickness of 4 mm as most adequate when using MTA as a root-end filling material.^21^ In a previous study by Rahimi et al,^22^ the microleakage with MTA as root-end filling material was not found to be significantly different among various thicknesses. Investigations of the sealing ability of CEM cement through dye penetration have revealed that the sealing properties of this root-end filling material parallel those of commercial types of MTA.^23^

The aim of this study was to compare the sealing ability associated with three different thicknesses of CEM cement as a root-end filling material in cavities prepared by ultrasonic retro-tips.

## Materials and Methods

Seventy single-rooted human premolar teeth extracted for periodontal or orthodontic purposes were selected for this study. The teeth were evaluated under stereomicroscope and radiography. Any teeth with caries, cracks, resorption, fractures, morphologic anomalies, and open apices were excluded from the study. The selected teeth were decoronated at cemento-enamel junction (CEJ) level with a diamond disk (D&Z, Darmstadt, Germany) under running water and air spray. Working length was determined with a #15 K-type file (Mani, Utsunomiya, Japan) 1 mm short of the apical foramen. The canals were prepared up to size #40 using the step-back technique and the shaping of the middle and coronal thirds was carried out by Gates Glidden burs 1, 2, and 3. During instrumentation procedures, root canals were irrigated with 10 ml of saline solution. All canals were obturated using gutta percha (Diadent, Korea) and AH-26 sealer (Dentsply, Konstanz, Germany) with the lateral compaction technique. The teeth were stored at 37°C and 100% humidity for 48 hours (Heratherm, Thermo Inc., Switzerland). The apical 3 mm of each tooth was resected perpendicular to the long axis of the tooth with a diamond bur under continuous water and air spray. Teeth were randomly divided into three experimental groups each containing 20 samples, and 5 positive and 5 negative controls.

In group 1, root-end cavities were prepared to a depth of 1mm perpendicular to the long axis using ultrasonic retrotips Kis-3D (Spartan, Missouri, USA).

In samples of groups 2 and 3, the same procedure was done to depths of 2 mm and 3 mm, respectively. In positive controls, the cavities were prepared to a depth of 3 mm.

The cavities were then irrigated and dried by paper cones. The CEM cement was mixed according to the manufacturer’s instructions on a sterile glass slab and filled into the cavities with the aid of a small condenser (Kerr Hawe, Orange, CA, USA). Any excess material was removed with a sterile cotton swab. The quality of the root-end fillings was confirmed by radiographs in two directions (mesiodistal and buccolingual), while the root-end cavities of controlled groups remained empty. In all experimental and positive control groups, two layers of nail varnish were applied to the surface of the teeth up to the level of the resected root-end. In negative controls, the entire surface of the teeth was covered with two layers of nail varnish.

All teeth were kept at 37°C and 100% humidity for 48 hours, and then immersed into synthetic tissue fluid (STF) with pH=7 for 48 hours. The specimens were placed horizontally in 2% Rhodamine B (Merk, Darmstadt, Germany) for 48 hours. Then the samples were rinsed for 10 min under running water. After that, two facial and lingual fissures were created along the long axis of the roots using a diamond disc and the roots were longitudinally resected into two mesial and distal halves.

The maximum amount of linear dye penetration was measured under a stereomicroscope (Zeiss, Munich, Germany) at ×16 magnification with 0.1 mm accuracy and using Image J software (35d; National Institutes of Health, USA).

The data were analyzed with one-way ANOVA analysis and the Bonferroni post-hoc test was utilized to reveal specific group differences. The level of significance was set at P < 0.05.

## Results

**Figure 1.**
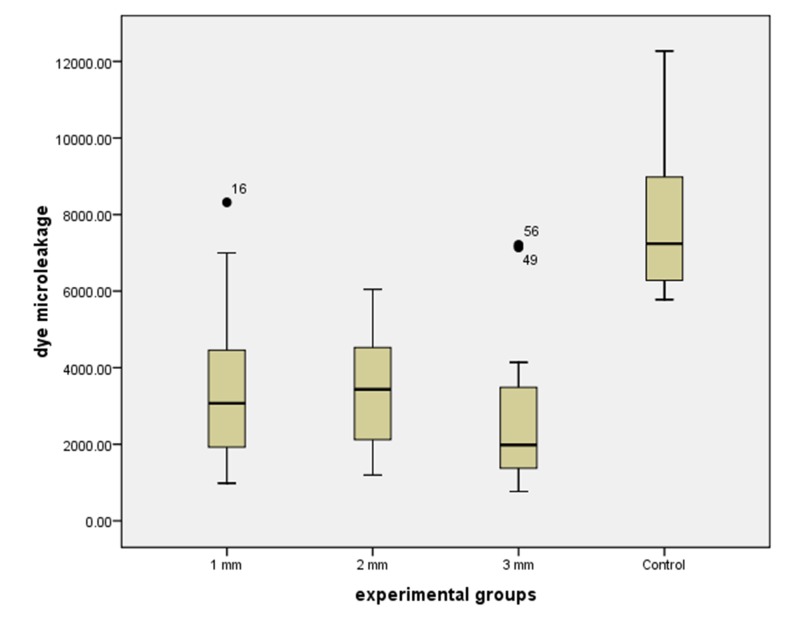
Dye microleakage amounts in experimental and control groups (µm).

No dye microleakage was noted in the negative control samples. The mean ± standard deviation of dye penetration in the 1, 2, and 3 mm groups were 3395.58 ± 1893.44, 3410.47 ± 1440.58, and 2581.65 ± 1852.90 micrometers, respectively (Figure 1).

Significant ‘Skewness’ and ‘Kurtosis’ tests confirmed non-normal data distribution. Natural logarithmic transformation provided normal distribution of the data. Analysis of variance indicated significant differences among the tested groups (P = 0.000). However, Bonferroni post-hoc test revealed that only the positive control group differed from the experimental groups (P = 0.000; Table 1). Consequently, there was no significant difference among sealing ability of 1, 2 and 3 mm CEM cement as a retrofilling material.

**Table 1:** Mean dye leakage (upper and lower 95% confidence interval) of experimental groups (micrometer)

	Groups		
Variable	1 mm	2 mm	3 mm
Leakage	3395.58 (4281.74-2509.42)	3410.47 (4084.68-2736.25)	2581.65 (3448.84-1714.47)

## Discussion

The success of periradicular surgery directly depends on the achievement of a good apical seal, utilizing a well-adapted root-end filling material that prevents the leakage of irritants from the root canal system into the periradicular region.^24^ In this study, the microleakage of three different thicknesses of CEM cement as a root-end filling material was evaluated by the dye penetration method. Findings indicated no significant differences among 1, 2, and 3 mm of CEM cement as a root end filling material. But in 1-mm and 2-mm depths, the leakage was more than the root-end filling material’s depth; in other words, only 3-mm depth was capable of providing adequate seal.

Apical microleakage has been assessed using various methods including dye/ink, bacterial/endotoxin leakage, radioisotope tracing, and fluid filtration technique.^25-31^ There is no evidence to favor the superiority of any particular method. However, the dye penetration technique is widely used for microleakage studies because dyes are cheap, safe, easily available, and also relatively easy to be stored, used and to have their penetration assessed quantitatively.^32,33^ Different dyes have been employed for dye penetration test as India ink and methylene blue. In this study, the extension of dye (2% Rhodamine B) penetration was used as the criteria for evaluation. The use of methylene blue in marginal sealing studies has been debated, due to its incompatibility with alkaline substances, which may induce discoloration of the dye.^34^ It is known that methylene blue dye presents an acid character and Rhodamine B, a basic one. Rhodamine B is a basic intense organic dye, soluble in water at room temperature, also solvent in alcohols and common organic solvents, in addition to being highly stable.^35^ Since color stability of organic dyes is an important factor that must be observed in microleakage studies, and because of alkali conditions around CEM cement, in this study Rhodamine B dye was used for leakage assessment.

An ideal root-end cavity preparation is a class I cavity at least 3 mm deep with parallel walls.^36^ Achieving this with the classical method in surgical endodontics is held back by several difficulties such as limited access, root anatomy, and tooth angulation. To avoid these problems, Piezoelectric ultrasonic devices for root-end preparation have been developed and are nowadays used as standard tools for retrograde cavity preparation.^37-39^

In another *in-vitro* study, the influence of the thickness of mineral trioxide aggregate on the sealing ability of root-end fillings was assessed by Valois et al.^21^ The 1-mm-thick MTA was the least effective in preventing apical leakage. No significant difference was found between 2- and 3-mm-thick MTA. Four-millimeter-thick MTA was found to be significantly more effective than the other thicknesses tested. These researchers suggested a thickness of 4 mm as most adequate when MTA is used as a root-end filling material.^21^ Similar findings were obtained in our study and CEM cement in 3-mm thickness present the most effective sealing ability.

The apical sealing ability of CEM cement has been reported to be similar to different commercial types of MTA.^23^ In a previous study, microleakage in the 3-mm and 2-mm root-end cavities was less than that in 1 mm depth cavities, but analysis of variance revealed no significant differences across the three different thicknesses.^22^ The current study led to similar results but with CEM cement used as root-end filling material.

## Conclusion

Based on the findings of this in vitro study, the CEM cement demonstrated adequate root-end sealing ability in 3-mm thickness.

## Acknowledgement

The authors would like to extend their appreciation to the Office of the Vice Chancellor for Research, Tabriz University of Medical Sciences, for the financial support of this study.
